# Adiposity and Long-Term Adiposity Change Are Associated with Incident Diabetes: A Prospective Cohort Study in Southwest China

**DOI:** 10.3390/ijerph182111481

**Published:** 2021-10-31

**Authors:** Yun Chen, Yiying Wang, Kelin Xu, Jie Zhou, Lisha Yu, Na Wang, Tao Liu, Chaowei Fu

**Affiliations:** 1 School of Public Health, Key Laboratory of Public Health Safety, NHC Key Laboratory of Health Technology Assessment, Fudan University, Shanghai 200032, China; 18211020001@fudan.edu.cn (Y.C.); xukelin@fudan.edu.cn (K.X.); na.wang@fudan.edu.cn (N.W.); 2 Guizhou Center for Disease Control and Prevention, Guiyang 550004, China; wyy123789123789@163.com (Y.W.); zhoujie19872014@163.com (J.Z.); 18785054757@163.com (L.Y.)

**Keywords:** general obesity, abdominal obesity, long-term adiposity change, type 2 diabetes, cohort study

## Abstract

In order to estimate the associations of different adiposity indicators and long-term adiposity changes with risk of incident type 2 diabetes (T2DM), we conducted a 10-year prospective cohort study of 7441 adults in Guizhou, China, from 2010 to 2020. Adiposity was measured at baseline and follow-up. Cox proportional hazard models were used to estimated hazard ratios (HRs) and 95% confidence intervals (95% CIs). A total of 764 new diabetes cases were identified over an average follow-up of 7.06 years. Adiposity indicators, body mass index (BMI), waist circumference (WC), waist-height ratio (WHtR), and long-term adiposity changes (both weight change and WC change) were significantly associated with an increased risk of T2DM (adjusted HRs: 1.16–1.48). Significant non-linear relationships were found between weight/WC change and incident T2DM. Compared with subjects with stable WC from baseline to follow-up visit, the subjects with WC gain ≥9 cm had a 1.61-fold greater risk of T2DM; those with WC loss had a 30% lower risk. Furthermore, the associations were stronger among participants aged 40 years or older, women, and Han Chinese. Preventing weight or WC gain and promoting maintenance of normal body weight or WC are important approaches for diabetes prevention, especially for the elderly, women, and Han Chinese.

## 1. Introduction

Diabetes is one of the leading causes of death and disability worldwide and affects more than 422 million people [1], while around 90% of patients have type 2 diabetes (T2DM). China has the highest number of people with diabetes (114.4 million) in the world, and 842,993 deaths are attributed to diabetes in 2017 [2]. Moreover, diabetes patients are at elevated risk of complications including diabetic nephropathy, cardiovascular disease, stroke, vision loss, and amputation of feet or legs [3]. The Global Burden of Disease (GBD) Study reported that high fast plasma glucose became China’s sixth-leading cause of disability-adjusted life-years (DALYs) with the attributable DALYs burden affecting 1802.3/100,000 of the population in 2016 [4]. Given the high prevalence and severity of diabetes, both determining the most common risk factors of diabetes and identifying individuals at high risk are vital for the prevention of diabetes as well as diabetes complications [5] and economic burden reduction [6]. 

There was growing evidence that T2DM was prevented or delayed by lifestyle modification, including a healthy diet, smoking cessation, a less sedentary lifestyle, and reducing obesity [6,7,8]. The GBD study indicated that high BMI scores is one of the most important risk factors for diabetes [4], which was consistent with previous epidemiological studies that have reported that obesity is associated with a higher risk of incident T2DM [9,10,11]. Assessment of obesity in populations has commonly relied on measures of body mass index (BMI) for general obesity [12] and waist circumference (WC) for abdominal obesity [13]. However, it has been suggested that long-term weight or WC change might be better than baseline BMI or WC for assessing the effect of excess fat, with it reflecting the individual differences in frame size and lean mass [14]. To our knowledge, few cohort studies have been conducted to assess the effect of weight change or change in body fat distribution on the risk for T2DM, and the benefits of weight loss were not consistent in previous observational studies [15,16,17]. Some studies have observed that weight loss significantly reduced the incidence of T2DM [17], while other studies have found little or no benefit [15,16]. However, most previous cohort studies were conducted in western countries where the prevalence of obesity was relatively higher compared to China [18]. Furthermore, the impact of change in WC on T2DM was likely to differ over races from country to country [19]. 

To date, we only found a cohort study among 10,419 Chinese adults with a mean follow-up of 2.8 years that had assessed the associations of adiposity and adiposity change with T2DM risk [20]. However, the relatively short follow-up period was not enough to understand a long-term adiposity change on the risk of incident T2DM. Furthermore, the dose–response relationship between long-term adiposity changes and incident T2DM remained unclear. Moreover, little is known with respect to whether associations between long-term adiposity changes and T2DM were inconsistent by different sociodemographic strata, such as age, sex, different ethnic groups, and duration of change. Exploring potential effect modifications will have important implications for clinical practice because such evidence may help to identify sensitive subpopulations and inform about effective targets for the prevention of T2DM, especially for the population in Southwest China with characteristics of being multi-ethnic, being relative backward in terms of economy and cultural environment, and scarcity in healthcare resources. In order to clarify these interests, large-scare prospective studies are needed to establish the associations between long-term adiposity changes and incident T2DM and to explore the potential differences in different population groups.

Therefore, based on the first 10-year prospective cohort study of adults in Southwest China, we aimed to test the hypothesis that baseline adiposity indicators and long-term body adiposity changes over time had independent effects on the risk of incident T2DM. Moreover, we aimed to examine possible interactions between long-term body adiposity changes and sociodemographic factors to determine whether long-term body adiposity changes have differential effects on incident T2DM in different populations.

## 2. Materials and Methods

### 2.1. Study Design and Population

The Guizhou Population Health Cohort Study (GPHCS) is a prospective community-based cohort in Guizhou province, China. Based on the multistage proportional stratified cluster sampling method, a total of 9280 adult residents from 48 townships of 12 districts in Guizhou province were recruited into this study from 2010 to 2012. The inclusion criteria were as follows: (1) aged 18 years or above; (2) living in the study region and having no plan to move out; (3) completing survey questionnaire and blood sampling; and (4) signing the written informed consent. This study was approved by the Institutional Review Board of Guizhou Province Centre for Disease Control and Prevention (No. S2017-02). All subjects provided the written informed consent at enrollment. 

Information was collected by trained investigators by using a structured questionnaire via a face-to-face interview. Baseline information included demographic characteristics (sex, age, ethnicity, education, marriage status, and occupation), lifestyle (smoking status, alcohol use, and physical activity), and history of chronic diseases. All participants were followed up for major chronic diseases and vital status by utilizing a repeated investigation during 2016–2020, and 1117 (12.04%) were lost to follow-up. All deaths were confirmed through the Death Registration Information System and Basic Public Health Service System. We further excluded 689 individuals with a history of T2DM at baseline, 19 missing T2DM status at follow-up, and 14 without height or weight information at baseline. Finally, the remaining 7441 participants were eligible for the analysis.

### 2.2. Anthropometric Measurements

Anthropometric measurements, including height, body weight, WC, and blood pressure, were measured by trained investigators. Standing height was measured to the nearest 0.1 cm using a portable stadiometer. Weight was measured to the nearest 0.1 kg by using a digital weighing scale. WC was measured to the nearest 0.1 cm at the midpoint between the lowest rib margin and the iliac crest. BMI was calculated as body weight in kilograms divided by square height in meters (kg/m^2^). BMI was evaluated with the following two methods: (1) per standard deviation (SD) increase; and (2) divided into four categories following the Chinese BMI classification standard (low normal weight < 22.0; high normal weight 22.0–23.9; overweight 24.0–27.9; and obese ≥ 28.0 kg/m^2^) [21]. WC was evaluated with the following two methods: (1) per SD increase; and (2) divided into two categories (normal weight < 85 cm in women and <90 cm in men; and abdominal obesity ≥ 85 cm in women and ≥90 cm in men) [20]. Waist-height ratio (WHtR) was calculated as WC in centimeters divided by height in centimeters and evaluated with the following two methods: (1) per SD increase; and (2) divided into two categories (normal weight < 0.5 and abdominal obesity ≥ 0.5) [20]. Participants’ weight change was calculated by recording weight at the follow-up minus the weight recorded at baseline and evaluated with the following two methods: (1) per SD increase; and (2) divided into four categories according to its distribution (loss of >2 kg, loss of ≤2 to gain of <2 kg, gain of ≥2 to gain of <6 kg, and gain of ≥6 kg). WC change was calculated by the WC at follow-up minus WC recorded at baseline and evaluated with the following two methods: (1) per SD increase; and (2) divided into four categories according to its distribution (loss of >3 cm, loss of ≤3 to gain of <3 cm, gain of ≥3 to gain of <9 cm, and gain of ≥9 cm).

### 2.3. Definitions

Venous blood samples were obtained from participants after at least 8 h overnight fast to measure fasting plasma glucose (FPG), total cholesterol (TC), triglycerides (TG), high-density lipoprotein cholesterol (HDL-C), and low-density lipoprotein cholesterol (LDL-C). A 2-h oral glucose tolerance test (OGTT) with 75 g of glucose was carried out for participants. T2DM was defined if participants met either of the following criteria: (1) self-reported doctor diagnosis of diabetes or use of anti-diabetic medications; (2) FPG ≥ 7.0 mmol/L; and (3) OGTT ≥ 11.1 mmol/L; (4) HbA1c ≥ 6.5% [22]. Impaired glucose regulation (IGR) was defined as follows: (1) FPG range from 5.6 to 6.9 mmol/L; (2) OGTT range from 7.8 to 11.0 mmol/L; and/or (3) HbA1c range from 5.7% to 6.4% [20]. Blood pressure was the average value of three measurements and measured to 0.1 mmHg by using the same model electronic sphygmomanometer. Hypertension was defined as follows: (1) self-reported doctor diagnosis of hypertension or use of hypertension medications; and/or (2) systolic blood pressure ≥ 140 mmHg and/or diastolic blood pressure ≥ 90 mmHg [23]. Dyslipidemia was determined if participants met any of the following criteria: (1) self-reported doctor diagnosis of dyslipidemia or use of lipid regulating drugs; (2) high TC: TC ≥ 6.22 mmol/L; (3) high TG: TG ≥ 2.26 mmol/L; (4) low HDL-C: HDL-C < 1.04 mmol/L; (5) and high LDL-C: LDL-C ≥ 4.14 mmol/L [24]. 

### 2.4. Statistics Analyses

The Student’s *t*-test for continuous variables and the Chi-square test for categorical variables were utilized in order to compare the differences between new T2DM cases and non-T2DM subjects. The person-years (PYs) of follow-up was calculated from the date of enrolling the cohort to the date of diagnosis of T2DM, death, or follow-up, whichever came first. Cox proportional hazards regression model was used to determine the associations of baseline general (BMI), abdominal (WC and WHtR) adiposity indicators, and long-term adiposity changes (weight change and WC change) with incident T2DM. We fitted three separate models for the association between each adiposity indicator and incident T2DM: (1) Model 1—adjusted for age (as continuous) and sex; (2) Model 2—Model 1 plus ethnicity (Han Chinese or non-Han Chinese: Han Chinese was from the largest ethnicity in China; non-Han Chinese was from ethnic minorities in China, including Bouyei, Miao, Dong, Yi, Tujia, and so on in this study.), education (<9 or ≥9 years), marriage status (married or other), occupation (farmer or other), smoking status (current smoker or non-smoker), alcohol use (yes or no), physical activity (yes or no), history of hypertension (yes or no), history of dyslipidemia (yes or no), and IGR (yes or no); (3) Model 3—Model 2 plus baseline BMI (in the analysis of WC, WHtR, and weight change) or baseline WC (in the analysis of WC change). In order to assess the robustness of the results, we repeated Model 3 after excluding the individuals who were followed up in less than 2 years. A multivariable Cox model with restricted cubic spline with 4 knots at the 5th, 35th, 65th, and 95th percentiles was applied to describe a potential curvilinear association of weight change or WC change with incident T2DM. We used the Schoenfeld residuals to test the assumption of hazard proportionality in Cox regression models and found no evidence of nonproportionality. 

We examined potential effect modification by age (<40 years old or ≥40 years old), sex, and ethnicity by first including multiplicative interaction terms in the multivariable Cox models and then fitting stratified models. All statistical tests were two-sided, and *p* < 0.05 was considered statistically significant. All analyses were performed in R software (Version 4.0.3; R Foundation for Statistical Computing, Vienna, Austria).

## 3. Results

### 3.1. Study Participants

**Figure 1:**
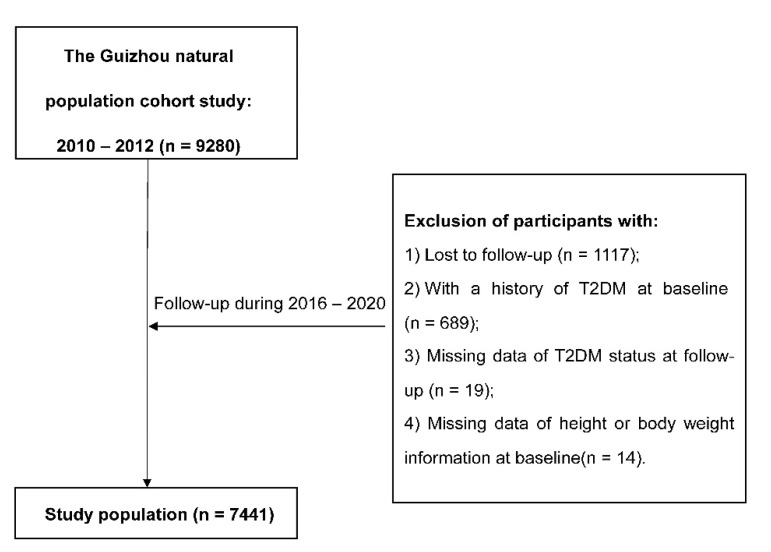
The flowchart.

After removing all participants with diabetes at baseline, a total of 7441 adults were eligible for the analysis (Figure 1), and the general characteristics of participants at baseline were presented in Table 1. Of all subjects, 47.0% were men, with a mean age of 43.97 ± 15.04 years old. During the follow-up, 764 new T2DM cases were identified. Compared with participants who remained free of T2DM, new T2DM cases were older and had higher baseline BMI, WC, and WHtR, and they were more likely to be farmers, to be current smokers, or to have a higher prevalence of hypertension, dyslipidemia, or IGR, and had a lower proportion of non-Han or education level. The average BMI, WC, and WHtR were higher at the follow-up compared with those at baseline (Table S1).

### 3.2. Associations between Baseline BMI, WC, and the WHtR with Incident T2DM

During the mean (SD) follow-up PYs of 7.06 (1.34), the crude incident density of T2DM was 14.54 per 1000 PYs. Baseline BMI, WC, and WHtR were associated with incident T2DM in the study population. As shown in Table 2, per SD increase in BMI was associated with a higher risk of incident T2DM (HR: 1.22; 95% CI: 1.16, 1.29), after adjustment for potential covariates. Compared with the normal weight group (BMI: 22.0–23.9 kg/m^2^), obese participants (BMI: ≥28.0 kg/m^2^) experienced an increased risk in incident T2DM with full adjusted HR of 2.37 (95% CI: 1.83, 3.05). As for per SD increase in WC and WHtR, the adjusted HRs (Model 2) for incident T2DM were 1.35 and 1.34, respectively. In the analyses of WC and WHtR, the associations were slightly attenuated after adjustment for baseline BMI but remained significant (Model 3). Participants with abdominal obesity (WC ≥85/90cm) or high WHtR (≥0.5) had significant increased HRs for incident T2DM compared with participants with normal WC or low WHtR (<0.5), which were 1.65 (95% CI: 1.33, 2.04) and 1.47 (95% CI: 1.23, 1.76), respectively, in Model 3.

### 3.3. Associations between Long-Term Adiposity Changes with Incident T2dm

**Figure 2:**
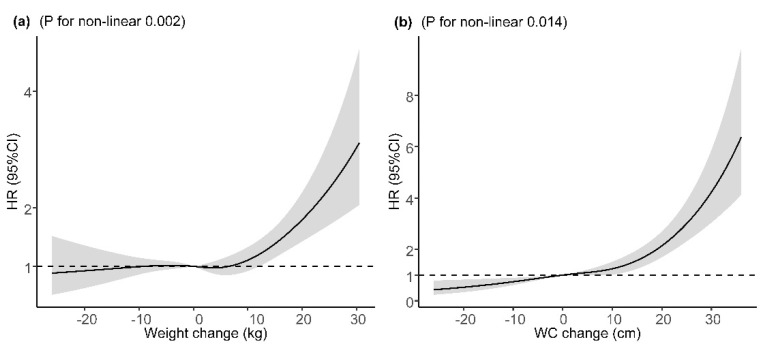
Dose–response relation between weight/WC change and incident T2DM. (**a**) Dose–response relation between weight change and incident T2DM; (**b**) dose–response relation between WC change and incident T2DM. Adjusted for age (continuous variable), sex, ethnicity, education, marriage, occupation, smoking status, alcohol use, physical activity, history of hypertension, history of dyslipidemia, IGR, and baseline BMI value (in the analyses of weight change) or baseline WC (in the analyses of WC change). HR, hazard ratio; 95% CI, 95% confidence interval; WC, weight circumference.

The mean (SD) change in weight was 2.10 (8.71) kg and 5.77 (10.09) cm in WC during the follow-up. Long-term weight change and WC change were found to be associated with increased risk of incident T2DM after adjustment for major covariates and baseline BMI or WC, with adjusted HRs of 1.16 (95% CI: 1.07, 1.26) and 1.48 (95% CI: 1.35, 1.62), per SD increase (Table 3). Compared with participants who maintained baseline WC (±3 cm), participants with a WC loss of >3 cm had a 30% lower risk of incident T2DM and those with a WC gain of ≥9 cm had a 61% higher risk. However, the association of weight gain of ≥6 kg with incident T2DM was not statistically significant after adjustment for major covariates and baseline BMI. In the sensitivity analysis, the corresponding effect estimates of baseline BMI, WC, WHtR, weight change, and WC change from baseline to the follow-up on incident T2DM did not change substantially after excluding participants who were diagnosed with T2DM within two years after entering the cohort (Figure S1). For the potential non-linear relationship, restricted cubic spline regressions demonstrated a significant J-shaped relationship between weight change or WC change with incident T2DM after adjustment for major covariates and baseline BMI or WC, with a steeper slope at a weight gain of ≥10 kg or a WC gain of 10 cm, respectively (Figure 2). 

### 3.4. Subgroup Analysis and Effect Modification

**Figure 3:**
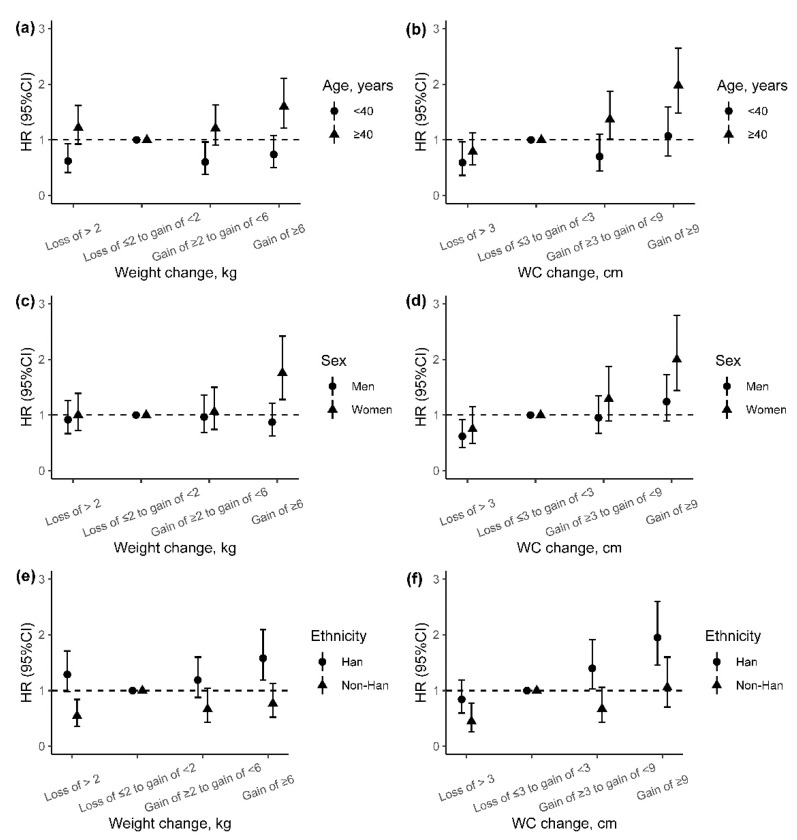
The incident risk of T2DM associated with adiposity changes from baseline to follow-up by age, sex, and ethnic group. (**a**,**b**) Association between weight or WC change with risk of incident T2DM by age; (**c**,**d**) association between weight or WC change with risk of incident T2DM by sex; (**e**,**f**) association between weight or WC change with risk of incident T2DM by ethnicity. Adjusted for age (continuous variable), sex, ethnicity, education, marriage, occupation, smoking status, alcohol use, physical activity, history of hypertension, history of dyslipidemia, IGR, and baseline BMI value (in the analyses of weight change) or baseline WC value (in the analyses of WC change). HR, hazard ratio; 95% CI, 95% confidence interval; WC, weight circumference.

We also explored the potential effect modification of baseline age, sex, and ethnicity on the association of adiposity change indicators with incident T2DM, although the differences of weight change and WC change between groups were almost not significant (Figure S2). We found higher adjusted HRs of incident T2DM for weight change and WC change among participants older than 40 years than those under 40 years (*p* for interaction: 0.008 and 0.019) (Figure 3a,b). In addition, the associations between weight change and incident T2DM were stronger among women and Han Chinese than men and non-Han Chinese (*p* for interaction: 0.025 and 0.005), especially for those with weight gain of ≥6 kg (Figure 3c–e). However, no effect modification on the association between WC change and incident T2DM was found for sex and ethnicity (Figure 3d–f). 

## 4. Discussion

Based on a prospective cohort study in Southwest China, we found that general and abdominal adiposity indicators were strongly associated with the risk of incident T2DM in adults, and the associations of the latter were stronger. Using longitudinal measures of weight and WC at baseline and follow-up, this study provided evidence that per SD increase in weight and WC changes were associated with a higher risk of T2DM. Restricted cubic splines suggested the non-linear relationships for weight change and WC change with incident T2DM. Furthermore, we found that weight and WC gain during midlife and later life (after 40 years of age) were important risk factors for T2DM. The observed associations were also stronger among women and Han Chinese than men and non-Han Chinese. 

Previous cohort studies or meta-analyses have demonstrated that adiposity indicators such as BMI, WC, and WHtR were the strongest modifiable risk factors of T2DM [10,20,25,26]. In this study, the HR of incident T2DM was increased with the increment of BMI, which was comparable with two cohort studies in China [10,20]. The presented study reported that the prevalence rate of abdominal obesity was higher than general obesity (13.2% vs. 6.3%). The abdominal adiposity indicators, indicating the degree of visceral adiposity, were proposed to be better predictors of T2DM [10,20,25]. Although the finding remains controversial [27,28], a meta-analysis based on 32 studies out of 432 publications reported that BMI, WC, and WHtR have similar associations with incident T2DM, and the pooled relative risks were equivalent for SD increase [26]. In this study, WC and WHtR were associated with a higher risk of incident T2DM than BMI (HR: 1.28–1.32 vs. 1.22), which attenuated but were still significant after additional adjustment of baseline BMI. 

A few cohort studies have linked long-term adiposity changes with incident T2DM, and their results were not consistent [15,20,29,30]. In the current analysis, we found that the risk of T2DM was positively associated with weight change and WC change and was stronger in WC change (HRs for per SD increase: 1.48 vs. 1.16). The findings that both the adiposity change indicators were associated with an excessive risk of T2DM were consistent with previous studies [20,29]. Compared with previous studies that reported no or higher effect of WC loss on T2DM risk [15,20], the present study has linked WC loss with lower T2DM risk over an average 7-year period, although it is not clear whether WC loss was caused by participants’ lifestyle changes or diseases. In this study, WC change showed a J-shape relation with T2DM, and it helped to infer that the risk of T2DM might decrease with WC loss. Different effects of WC loss observed in this study may be caused by the inconsistency of demography characteristics [15] and duration of follow-up time [20]. Subjects with WC gain ≥9 cm had a higher HR for incident T2DM compared with those who had stable WC, which was consistent with previous studies [20,30,31,32]. A 9-year follow-up study based on the Insulin Resistance Syndrome cohort demonstrated that WC gain was an important risk factor for T2DM in subjects with prediabetes [31]. Studies in general participants also reported that WC gain was associated with an excessive risk of T2DM [20,30,32]. In addition, we did not observe an increase in T2DM risk associated with weight gain in the entire cohort, although such association was significant in a subgroup, such as the elderly, women, and Han Chinese. Thus, we inferred that WC change was more sensitive than weight change with respect to reflecting the risk of T2DM increased by adiposity change in the present study. The results may be explained by WC being more associated with visceral fat, which has strong association with insulin resistance. The excess visceral fat results in inflammation in visceral fat, which may worsen insulin resistance [33]. Meanwhile, WC not only links to visceral fat but also to subcutaneous abdominal fat, and the expansion of subcutaneous cells results in excessive ectopic lipid accumulation, which also had a strong association with insulin resistance [34].

The presence of effect modification by age has been reported for the association between adiposity and T2DM risk, and age modification on the association between adiposity change and risk of T2DM was also significant in this study. The HR estimates associated with adiposity change indicators were appreciably higher in individuals aged 40 years or above compared with those younger than 40 years. The finding that weight or WC loss in early life (under 40 years) was associated with decreased risk of T2DM was consistent with several previous reports [32,35]. This contrasts with the results that we did not observe a reduction in T2DM risk associated with measured weight or WC loss in midlife or later life. One explanation for the effect modification by age is that elderly adults may lose proportionately more muscle mass with weight or WC loss than younger ones [36], and the loss of skeletal muscle mass may decrease insulin sensitivity, which may negate the benefit derived from fat loss [37]. This study also found that weight or WC gain in midlife or later life may result in a significantly increased risk of T2DM, but weight or WC gain in early life does not. These findings highlight the importance of weight management during midlife and later life. 

In addition, the association between weight change and risk of incident T2DM also differed by sex and ethnicity, and it was stronger among women and Han Chinese. Women have a higher risk of T2DM compared with men due to higher body fat composition [38,39], more circulating FFA, and higher intramyocellular lipid content, all of which are factors predicted to promote insulin resistance [40]. The difference also is the consequence of the action of sex chromosomes and sex-specific hormones, including estrogens and progesterone [41]. The observed effect modification by the ethnic group may be due to genetic variation. Miao and Bouyei ethnicity were the two main minorities in this study population. These minorities had been reported to exhibit significant differences in their genetic background compared with Han Chinese [42]. Another reasonable explanation for the differences might be the residual confounding effects by other lifestyle factors such as dietary habits [43] for which we have not been able to control completely in this study. Thus, future work with a larger sample size is needed to confirm the effect modification of ethnic groups on the association between adiposity change indicators and risk of T2DM and to understand the possible mechanisms. 

Based on the first 10-year well-characterized population-based cohort in Southwest China, there were several strengths in this study. Firstly, relatively low loss to follow-up limited the potential bias for risk estimates. Secondly, we used a standardized measurement of different adiposity indicators rather than self-report and also assessed the weight change and WC change from baseline to follow-up. Therefore, exposure measurement errors were largely reduced. Finally, to the best of our knowledge, this is the first report on adiposity indicators and long-term adiposity changes in association with T2DM between different age, sex, and ethnic groups in Southwest China. Our results indicated the sensitive subpopulations and could result in clinical implications for target intervention in primary healthcare, such as adiposity management.

This study also had notable limitations. Firstly, only baseline information of most covariates was used in all analyses, which might result in residual confounding if those covariates may be time-varying. Secondly, even though current analyses adjusted for major potential confounding factors, residual confounding resulting from dietary factors cannot be excluded. Thirdly, because of only one follow-up, the timing of the onset of T2DM in this cohort could be inaccurate. Since there is a lack of information of OGTT and Hb1Ac in the follow-up visit, the T2DM incidence rate could be underestimated. Finally, adiposity was measured by indirect parameters, and a future better-designed study is required for confirming those findings from this study.

## 5. Conclusions

In conclusion, general obesity, abdominal obesity, weight gain, and WC gain significantly increased the risk of incident T2DM, and WC has a stronger association than BMI. The risk for incident diabetes increased with weight gain or WC gain, especially among the elderly, women, and Han Chinese. Our results contributed new evidence on the relationships of long-term adiposity change indicators with incident diabetes risk in adults of Southwest, China, and highlighted the need for focused attention on adiposity management and diabetes prevention, especially for the elderly, women, and Han Chinese. 

## Figures and Tables

**Table 1:** General characteristics of the study population at baseline in Southwest China.

Characteristics	Total	Non-T2DM	New T2DM	*p* value
Participants, *n*	7441	6677	764	
Age at baseline, years	43.97 ± 15.04	43.55 ± 15.09	47.62 ± 14.10	<0.001
Men, %	3494 (47.0)	3123 (46.8)	371 (48.6)	0.368
Non-Han Chinese, %	3057 (41.1)	2790 (41.8)	267 (34.9)	<0.001
Education ≥9 years, %	3177 (42.7)	2908 (43.6)	269 (35.2)	<0.001
Married, %	5979 (80.4)	5362 (80.3)	617 (80.8)	0.802
Farmer, %	4254 (57.2)	3777 (56.6)	477 (62.4)	0.002
Current smoker, %	2116 (28.4)	1874 (28.1)	242 (31.7)	0.040
Alcohol use, %	2354 (31.6)	2097 (31.4)	257 (33.6)	0.224
Physical activity, %	6458 (86.8)	5787 (86.7)	671 (87.8)	0.402
History of hypertension, %	1816 (24.4)	1591 (23.8)	225 (29.5)	0.001
History of dyslipidemia, %	4170 (56.0)	3705 (55.5)	465 (60.9)	0.005
IGR, % ^†^	2948 (39.6)	2612 (39.1)	336 (44.0)	0.010
BMI, kg/m^2^	22.74 ± 3.29	22.65 ± 3.23	23.53 ± 3.73	<0.001
<22.0	3407 (45.8)	3115 (46.7)	292 (38.2)	<0.001
22.0–23.9	1783 (24.0)	1607 (24.1)	176 (23.0)	
24.0–27.9	1781 (23.9)	1583 (23.7)	198 (25.9)	
≥28.0	470 (6.3)	372 (5.6)	98 (12.8)	
WC, cm ^†^	76.15 ± 9.26	75.84 ± 9.08	78.78 ± 10.33	<0.001
≥85/90	917 (13.2)	755 (12.1)	162 (22.2)	<0.001
WHtR ^†^	0.49 ± 0.06	0.48 ± 0.06	0.50 ± 0.07	<0.001
≥0.5	2573 (37.0)	2211 (35.6)	362 (49.5)	<0.001

^†^ Missing value. IGR, impaired glucose regulation; BMI, body mass index; WC, waist circumference; WHtR, waist-height ratio.

**Table 2:** The incident risk of T2DM associated with baseline BMI, WC, and the WHtR.

Characteristics	Cases, *n*	Incident Density/1000 PYs	HR (95% CI)		
			Model 1	Model 2	Model 3
BMI (per SD increase, kg/m^2^)	764	14.54	1.23 (1.16, 1.29) ***	1.22 (1.16, 1.29) ***	
<22.0	292	14.06	0.86 (0.71, 1.04)	0.85 (0.70, 1.02)	
22.0–23.9	176	12.02	1.00	1.00	
24.0–27.9	198	15.78	1.10 (0.90, 1.35)	1.13 (0.92, 1.39)	
≥28.0	98	30.71	2.32 (1.81, 2.98) ***	2.37 (1.83, 3.05) ***	
WC (per SD increase, cm)	731	14.75	1.33 (1.24, 1.42) ***	1.35 (1.26, 1.45) ***	1.28 (1.16, 1.42) ***
<85/90	569	13.18	1.00	1.00	1.00
≥85/90	162	25.42	1.94 (1.63, 2.31) ***	2.00 (1.67, 2.40) ***	1.65 (1.33, 2.04) ***
WHtR (per SD increase)	731	14.75	1.34 (1.25, 1.43) ***	1.34 (1.25, 1.43) ***	1.32 (1.18, 1.48) ***
<0.5	369	11.72	1.00	1.00	1.00
≥0.5	362	20.03	1.70 (1.46, 1.97) ***	1.72 (1.47, 2.00) ***	1.47 (1.23, 1.76) ***

Model 1: adjusted for age (continuous variable) and sex. Model 2: model 1 plus ethnicity, education, marriage, occupation, smoking status, alcohol use, physical activity, history of hypertension, history of dyslipidemia, and IGR. Model 3: model 2 plus baseline BMI value (in the analyses of WC and WHtR). ***: *p* < 0.001. PY, person-years; HR, hazard ratio; 95% CI, 95% confidence interval; BMI, body mass index; SD, standard deviation; WC, waist circumference; WHtR, waist-height ratio.

**Table 3:** The incident risk of T2DM associated with weight change and WC change from baseline to follow-up.

Characteristics	Cases, *n*	Incident Density/1000 PYs	HR (95% CI)		
			Model 1	Model 2	Model 3
Weight change (per SD increase, kg)	604	15.18	1.09 (1.01, 1.18) *	1.08 (1.00, 1.16)	1.16 (1.07, 1.26) ***
loss of >2	163	16.50	1.05 (0.84, 1.32)	1.06 (0.84, 1.33)	0.98 (0.78, 1.23)
loss of ≤2 to gain of <2	137	13.95	1.00	1.00	1.00
gain of ≥2 to gain of <6	120	13.43	0.96 (0.75, 1.23)	0.96 (0.75, 1.22)	0.96 (0.75, 1.23)
gain of ≥6	184	16.52	1.15 (0.92, 1.44)	1.13 (0.90, 1.42)	1.23 (0.98, 1.55)
WC change (per SD increase, cm)	571	15.69	1.15 (1.06, 1.25) **	1.15 (1.06, 1.24) **	1.48 (1.35, 1.62) ***
loss of >3	87	15.18	0.94 (0.71, 1.25)	0.93 (0.70, 1.24)	0.70 (0.52, 0.93) *
loss of ≤3 to gain of <3	113	14.06	1.00	1.00	1.00
gain of ≥3 to gain of <9	132	14.30	0.97 (0.76, 1.25)	0.97 (0.75, 1.24)	1.10 (0.86, 1.42)
gain of ≥9	239	17.85	1.21 (0.97, 1.52)	1.18 (0.95, 1.48)	1.61 (1.27, 2.03) ***

Model 1: adjusted for age (continuous variable) and sex. Model 2: model 1 plus ethnicity, education, marriage, occupation, smoking status, alcohol use, physical activity, history of hypertension, history of dyslipidemia, and IGR. Model 3: model 2 plus baseline BMI value (in the analyses of weight change) or baseline WC (in the analyses of WC change). ***: *p* < 0.001, **: *p* < 0.01, and *: *p* < 0.05; PY, person-years; HR, hazard ratio; 95% CI, 95% confidence interval; SD, standard deviation; WC, waist circumference.

## Data Availability

The raw data supporting the conclusions of this article will be made available by the authors upon reasonable request.

## Supplementary Materials

The following are available online at https://www.mdpi.com/article/10.3390/ijerph182111481/s1. Including one table and two figures: Table S1: General characteristics of the study population at follow-up in Southwest China; Figure S1: Sensitivity analysis after excluding participants who were diagnosed with T2DM within two years; Figure S2: Weight and WC change according to age, sex, and ethnicity.
