# Supplementary material for: Adiposity and Long-Term Adiposity Change Are Associated with Incident Diabetes: A Prospective Cohort Study in Southwest China
Source: Int J Environ Res Public Health. 2021 Oct 31;18(21):11481. doi: 10.3390/ijerph182111481 (PMC8582792; doi:10.3390/ijerph182111481)
Supplement: Supplementary file 1 [file ijerph-18-11481-s001.zip › ijerph-1430203-supplementary.pdf]

**Table S1. General characteristics of the study population at follow-up in Southwest China.**

| Characteristics | Total | Non-T2DM | New T2DM | P value |
|---|---|---|---|---|
| Participants, n | 7441 | 6677 | 764 | |
| Age at follow-up, years | 51.03 ± 14.97 | 50.60 ± 15.01 | 54.78 ± 14.09 | <0.001 |
| Men, % | 3494 (47.0) | 3123 (46.8) | 371 (48.6) | 0.368 |
| Non-Han Chinese, % | 3057 (41.1) | 2790 (41.8) | 267 (34.9) | <0.001 |
| Education ≥9 years, % | 3177 (42.7) | 2908 (43.6) | 269 (35.2) | <0.001 |
| Married, % | 6498 (87.3) | 5838 (87.4) | 660 (86.4) | 0.443 |
| Farmer, % | 2746 (36.9) | 2480 (37.1) | 266 (34.8) | 0.222 |
| Current smoker, % † | 1922 (27.8) | 1673 (27.2) | 249 (33.4) | <0.001 |
| Alcohol use, % † | 2334 (33.9) | 2052 (33.4) | 282 (37.9) | 0.017 |
| Physical activity, % † | 5666 (82.4) | 5053 (82.4) | 613 (82.3) | 0.953 |
| History of hypertension, % † | 2944 (41.6) | 2493 (39.4) | 451 (60.1) | <0.001 |
| History of dyslipidemia, % † | 5005 (52.9) | 1864 (50.5) | 365 (69.7) | <0.001 |
| IGR, % † | 2850 (51.1) | 2174 (44.6) | 676 (96.2) | <0.001 |
| BMI, kg/m$^2$ † | 23.56 ± 3.55 | 23.43 ± 3.44 | 24.65 ± 4.25 | <0.001 |
| <22.0 | 2042 (35.6) | 1869 (36.5) | 173 (28.0) | <0.001 |
| 22.0-23.9 | 1429 (24.9) | 1312 (25.6) | 117 (19.0) | |
| 24.0-27.9 | 1688 (29.4) | 1480 (28.9) | 208 (33.7) | |
| ≥28.0 | 585 (10.2) | 466 (9.1) | 119 (19.3) | |
| WC, cm † | 82.29 ± 9.54 | 81.82 ± 9.26 | 86.14 ± 10.90 | <0.001 |
| ≥85/90 | 1628 (29.3) | 1362 (27.5) | 266 (44.3) | <0.001 |
| WHtR † | 0.52 ± 0.06 | 0.52 ± 0.06 | 0.55 ± 0.07 | <0.001 |
| ≥0.5 | 3528 (63.6) | 3069 (62.1) | 459 (76.4) | <0.001 |

† missing value. IGR, impaired glucose regulation; BMI, body mass index; WC, waist circumference; WHtR, waist-height ratio.

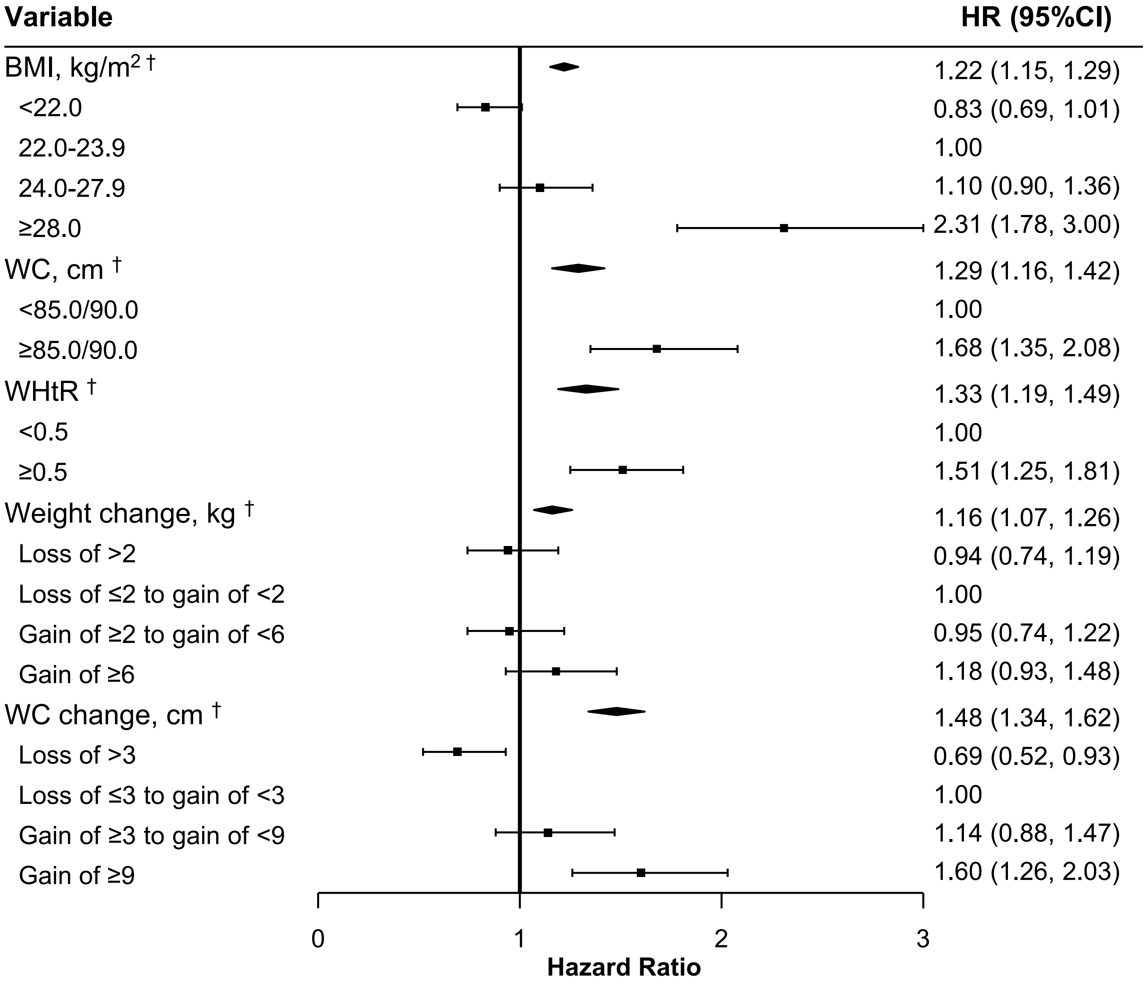


**Figure S1. Sensitivity analysis after excluding participants who were diagnosed with T2DM within two years.** Adjusted for age (continuous variable), sex, ethnicity, education, marriage, occupation, smoking status, alcohol use, physical activity, history of hypertension, history of dyslipidemia, IGR, and baseline BMI value (in the analyses of WC, WHtR, weight change) or baseline WC (in the analyses of WC change). † per SD change. HR, hazard ratio; 95%CI, 95% confidence interval; BMI, body mass index; WC, waist circumference; WHtR, waist-height ratio.

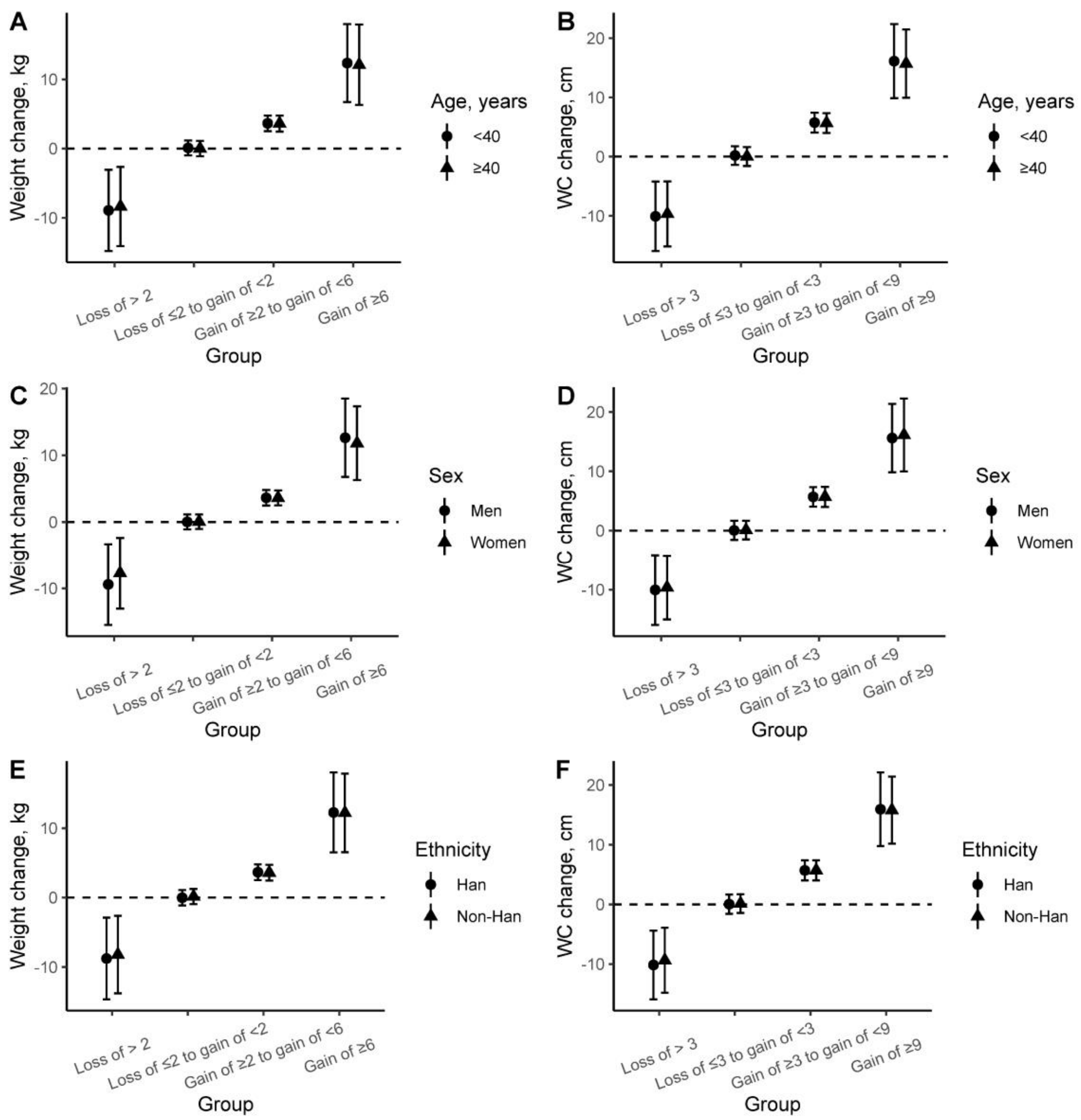


**Figure S2. The weight and WC change according to age, sex, and ethnicity.** WC, waist circumference.
